# Exposure to environmental factors increases connectivity between symptom domains in the psychopathology network

**DOI:** 10.1186/s12888-016-0935-1

**Published:** 2016-07-08

**Authors:** Sinan Guloksuz, Martine van Nierop, Maarten Bak, Ron de Graaf, Margreet ten Have, Saskia van Dorsselaer, Nicole Gunther, Roselind Lieb, Ruud van Winkel, Hans-Ulrich Wittchen, Jim van Os

**Affiliations:** Department of Psychiatry and Psychology, Maastricht University Medical Centre, P.O. BOX 616, 6200 MD Maastricht, The Netherlands; Department of Psychiatry, Yale University School of Medicine, New Haven, CT USA; Netherlands Institute of Mental Health and Addiction, Utrecht, The Netherlands; School of Psychology, Open University, Heerlen, The Netherlands; Department of Psychology, Division of Clinical Psychology and Epidemiology, University of Basel, Basel, Switzerland; University Psychiatric Center Katholieke Universiteit Leuven, campus Kortenberg, Leuvensesteenweg, Kortenberg, Belgium; Institute of Clinical Psychology and Psychotherapy, Technische Universität Dresden, Dresden, Germany; Max Planck Institute of Psychiatry, Munich, Germany; King’s College London, King’s Health Partners, Department of Psychosis Studies, Institute of Psychiatry, London, UK

**Keywords:** Psychopathology, Psychosis, Depression, Environment, Epidemiology

## Abstract

**Background:**

We investigated to what degree environmental exposure (childhood trauma, urbanicity, cannabis use, and discrimination) impacts symptom connectivity using both continuous and categorical measures of psychopathology.

**Methods:**

Outcomes were continuous symptom dimensions of self-reported psychopathology using the Self-report Symptom Checklist-90-R in 3021 participants from The Early Developmental Stages of the Psychopathology (EDSP) study and binary DSM-III-R categories of mental disorders and a binary measure of psychotic symptoms in 7076 participants from The Netherlands Mental Health Survey and Incidence Study (NEMESIS-1). For each symptom dimension in the EDSP and mental disorder in the NEMESIS-1 as the dependent variable, regression analyses were carried out including each of the remaining symptom dimensions/mental disorders and its interaction with cumulative environmental risk load (the sum score of environmental exposures) as independent variables.

**Results:**

All symptom dimensions in the EDSP and related diagnostic categories in the NEMESIS-1 were strongly associated with each other, and environmental exposures increased the degree of symptom connectivity in the networks in both cohorts.

**Conclusions:**

Our findings showing strong connectivity across symptom dimensions and related binary diagnostic constructs in two independent population cohorts provide further evidence for the conceptualization of psychopathology as a contextually sensitive network of mutually interacting symptoms.

## Background

Psychopathology traditionally is represented as categories that implicitly refer to nosological entities (e.g. Major Depressive Disorder) or trans-diagnostic symptom dimensions based on symptom-level clustering (e.g. severity of depressive symptoms). Vinogradov and colleagues [1] proposed an associationist model of the symptom dimension of paranoia and suggested that the origins of psychopathology may lie in a network of mental states giving rise to acute phase transitions that Odgers and colleagues showed can be modelled as part of a dynamic system [2]. According to recent elaborations and extensions of this model, psychopathology can be conceptualized as a network of causally linked sets of symptoms, which reciprocally impact on each other over time to progress toward a more distinct syndrome forged through a specific pattern of a dynamic network for each syndrome [3–6].

The conceptualization of psychopathology as a dynamic network of symptoms may provide a particularly useful tool to understand pathways to mental illness. For example, transition of a false belief to a crystallized delusion can be modelled as a vicious cycle composed of interacting steps, in which an initial paranoid state, arising out of malformed hyperconnectivity among temporally contiguous perceptions, becomes fixated through perpetuation of an internally generated maladaptive schema to explain odd experiences [1]. Hyperconnectivity may also expand through the network across symptom domains, triggering a chain reaction, wherein the primary false belief (e.g. persecutory delusional ideation) may provoke anxiety under certain circumstances (e.g. being in public), which in turn leads to a misperception of external stimuli (illusions and hallucinations) that creates added confusion and anxiety which further impairs logical thought process, and solidifies delusionary explanation that finally becomes a fixed delusion.

Recent findings in large general population cohort studies suggest that exposure to environmental risk factors (childhood trauma, urbanicity, cannabis use, and discrimination) reinforces connectivity between symptoms of affective dysregulation and psychosis expression in a dose response fashion, in accordance with the theory of environment-induced disturbances spreading through the psychopathology network, increasing psychosis admixture, and progressively expanding and reinforcing connectivity that ultimately gives rise to transition to a more severe, distinct clinical syndrome requiring medical care [7–12].

If the environment impacts on the connection between psychosis and affective dysregulation, the question rises to what degree this is specific for these two domains of psychopathology. An alternative explanation is that the environment broadly impacts on the connectivity between domains of psychopathology. The emergence of psychosis may then be seen as an indicator of general severity of increasingly connected psychopathology, rather than a specific illness category [9, 13–15]. In the light of these findings, we hypothesize that the degree of exposure to environmental factors that are known to be associated with mental health would increase the extent of connectivity between symptom dimensions. Therefore, the aim was to investigate to what degree environmental exposure impacts on symptom connectivity analysing measures of psychopathology in two independent, general population datasets. In order to examine to what degree the findings would be stable across categorically and dimensionally defined psychopathology, connectivity was examined at the level of both continuous dimensions of self-reported psychopathology using the Self-report Symptom Checklist-90-R (SCL-90-R) and binary DSM-III-R categories of mental disorders and a binary measure of psychotic symptoms.

## Methods

### Samples

#### The Early Developmental Stages of the Psychopathology (EDSP) study

The EDSP study collected information on the prevalence, incidence, risk factors, comorbidity and course of mental symptoms and syndromes in a random representative population sample of 3021 adolescents and young adults living in the Munich area (aged 14–24 years at baseline) at 4 waves: at baseline (T0e), and at 3 follow-ups after on average 1.6 (T1e), 3.5 (T2e), and 8.4 years (T3e), respectively. Details were provided elsewhere [16–18].

#### The Netherlands Mental Health Survey and Incidence Study (NEMESIS-1)

The NEMESIS-1 study collected information on the prevalence, incidence, and course of mental disorders in a random representative general population sample of 7076 at 3 waves: at baseline (T0n), at the 12-month follow-up (T1n), and at the 36-month follow-up (T2n). Details were provided elsewhere [19].

The EDSP project has been approved by the Ethics Committee of the Faculty of the Technische Universitat Dresden (No. EK-13811). The NEMESIS-1 was conducted with the approval of the ethics committee of the Netherlands Institute of Mental Health and Addiction, Utrecht, the Netherlands. All respondents provided informed consent.

## Instruments

### Assessment of psychopathology

#### EDSP

At each time point, participants completed the SCL-90-R, a comprehensive self-report symptom inventory, multidimensional in nature, and oriented to screen for a broad range of psychological problems and psychopathology in community respondents and respondents with somatic and mental disorders. The SCL-90-R contains 90 items, scored on a 5-point severity scale, measuring 9 primary symptom dimensions named “somatization,” “obsessive-compulsive,” (OC) “interpersonal sensitivity,” “depression,” “anxiety,” “hostility”, “phobic anxiety,” “paranoid ideation,” and “psychoticism.” Reliability and validity of the SCL-90-R are quite satisfactory [20]. The time frame is the past 2 weeks.

Consistent with previous analyses in this sample, the paranoid ideation and psychoticism dimensions were combined to create a single “psychosis” dimension [9, 21, 22].

#### NEMESIS-1

The CIDI version 1.1 (computerized version) [23] was used to diagnose DSM III-R mental disorders. The CIDI is a structured interview developed by the World Health Organization and has been found to have high inter-rater reliability and high test–retest reliability for most common mental diagnoses [24]. The following diagnoses were included in the current analysis: manic episode, major depression, dysthymia and any anxiety disorder (panic disorder, agoraphobia, simple phobia, social phobia, generalized anxiety disorder, and obsessive–compulsive disorder). Diagnoses represent lifetime presence at baseline and interval presence at follow-up interviews.

In addition, a binary measure of any clinically relevant psychotic symptom [25] was used as a measure of psychosis [26]. Previous studies using the CIDI showed that the instrument a reliable and valid assessment of mental disorders, with the exception of psychotic disorders [27]. The CIDI psychosis section consists of 17 psychosis items concerning delusions (13 items) and hallucinations (4 items). Each item is scored on a 1–6 scale: ‶1,″ no symptom; ‶2,″ psychotic symptom present but not clinically relevant; ‶3,″ psychotic symptom result of drug use; ‶4,″ psychotic symptom result of somatic disease; ‶5,″ true psychotic symptom; and ‶6,″ possible plausible explanation for what appears to be a psychotic symptom. Each participant with a score of 2, 5, or 6 was followed up for validation. Individuals endorsing at least one lifetime psychotic symptom were interviewed using questions from the Structured Clinical Interview for Diagnostic and Statistical Manual of Mental Disorders, Fourth Edition (SCID-I) over the telephone by an experienced clinician [28].

### Assessment of Trauma

#### EDSP

Self-reported lifetime (baseline) and interval (follow-up) exposure to trauma was assessed using the N-section of the DIA-X/M-CIDI on trauma and PTSD comprising 9 groups of specific traumatic events (presented by a respondent list) such as “experienced physical threat,” “experienced serious accident,” or “being sexually abused as a child.” Visual presentation of the list allowed respondents and interviewers to avoid speaking about sometimes embarrassing and stigmatising trauma by simply indicating the number of the event. Consistent with earlier analyses [14, 29], positive responses to any of the events were coded as “self-reported trauma.”

#### NEMESIS-1

Subjects were asked, using a semi-structured interview, whether they had experienced any kind of emotional, physical, psychological or sexual abuse before age 16 years, providing examples for each type of trauma. Subjects answered ‘yes’ or ‘no’ to each of the questions and were asked to give an indication about the frequency on a six-point scale: 1, never; 2, once; 3, sometimes; 4, regular; 5, often; 6, very often. Consistent with previous work [30], in the analyses, experience of trauma was a priori dichotomized as follows: ‘no early trauma’ if the score on any item was ≤3, and ‘early trauma’ if the score on any item was >3.

### Assessment of cannabis use

#### EDSP

Cannabis use was assessed with the L-section of the DIA-X/M-CIDI using the question “Have you ever used cannabis five times or more?” to define cannabis exposure. Conforming to previous work [22, 31], the DIA-X/M-CIDI cut-off of use of five times or more was used to define the binary variable for cannabis exposure.

#### NEMESIS-1

Consistent with previous work [32], the l-section of the CIDI 1.1 was used to define use of cannabis. In keeping with the previous work, “cannabis use,” was defined as use of cannabis a least once in the lifetime [32, 33].

### Assessment of urbanicity

#### EDSP

In agreement with earlier work [14, 34], urbanicity was defined as living, at baseline, in the urban region of the German city of Munich versus the surrounding areas of Munich. The urban area, hence defined, had a population density of 4061 persons per square mile; for the rural area, this was 553 persons per square mile.

#### NEMESIS-1

Guided by previous work [28, 35], the original five-level classification of the urbanization measure expressed as density of addresses per km^2^ was dichotomized as follows: levels 1 (<500), 2 (500–900) and 3 (1000–1499) were coded as 0 and levels 4 (1500–2499) and 5 (≥2500) as 1.

### Assessment of discrimination

#### NEMESIS-1

Perceived discrimination was assessed only in the NEMESIS-1 and not in EDSP. At baseline, participants were asked if they had experienced discrimination over the past year because of their skin colour or ethnicity; gender; age; appearance; disability; or sexual orientation [11]. Experienced discrimination was dichotomized as follows: ‘experienced discrimination’ if the participant answered one or more of the questions with “Yes”, and ‘no discrimination’ if the participant answered all questions with “No” [36].

### Statistical analysis

All analyses were carried out with Stata 13.1 [37].

### EDSP

Given the fact that outcome was measured at each time point, and in keeping with previous work [14, 38], data were analysed in the “long format” [each individual contributing 4 observations (T0e, T1e, T2e, and T3e)]. The analyses, therefore, were cross-sectional. Multilevel regression models using the XTREG command were applied to analyse whether the association between symptom dimensions increased as a function of the extent of exposure to environmental risk factors (continuous variable: from 0 = no exposure, to 3 = exposure to all environmental factors), hereafter referred to as ‘environmental exposure score’. Therefore, for each symptom dimension as the dependent variable, analyses were carried out including, in separate models, each of the 7 other symptom dimensions, and its interaction with environmental exposure score as independent variables. In order to correct for the clustering of multiple observations within subjects, all analyses were controlled by adding subject ID to the models as random intercepts.

### NEMESIS-1

Given the fact that outcomes were similarly measured at each time point, data were analysed in the “long format” [each individual contributing 3 observations (T0n, T1n, T2n)]. To calculate the statistical interaction under an additive model (similar to the EDSP analyses described above), the BINREG procedure, which fits generalized linear models for the binomial family estimating risk differences, was used to model interactions between environmental exposure score (the categories of 3 and 4 exposures were merged due to small numbers in the latter to create a continuous variable: from 0 = no exposure, to 3 = exposure to ≥3 environmental factors) and diagnostic domains. Therefore, for each of the mental disorders as the dependent variable, analyses were carried out including each of the other 4 diagnostic domains and its interaction with environmental exposure score as independent variables. In order to correct for clustering of multiple observations within subjects, all analyses were controlled by adding subject ID to the models as random intercepts.

## Results

Characteristics of the study populations at different time points are shown in Tables 1 and 2.

**Table 1:** Characteristics of the population at different time points (EDSP)

	T0e (*n* = 3021)	T1e (*n* = 1228)*	T2e (*n* = 2548)	T3e (*n* = 2210)
	*M* (SD)	*M* (SD)	*M* (SD)	*M* (SD)
Sex (male)^a^	1533 (50.74)	637 (51.87)	1297 (50.90)	1135 (51.36)
Age	18.26 (3.34)	16.72 (1.19)	21.74 (3.39)	26.62 (3.47)
Symptom dimension scores^b^				
Anxiety	1.33 (0.37)	1.22 (0.32)	1.19 (0.28)	1.17 (0.31)
Depression	1.43 (0.46)	1.31 (0.39)	1.32 (0.39)	1.30 (0.41)
Hostility	1.45 (0.50)	1.34 (0.45)	1.29 (0.40)	1.24 (0.35)
Interpersonal sensitivity	1.48 (0.49)	1.33 (0.43)	1.35 (0.43)	1.28 (0.39)
Obsessive-compulsive	1.47 (0.44)	1.36 (0.40)	1.35 (0.38)	1.32 (0.38)
Phobic anxiety	1.24 (0.30)	1.17 (0.26)	1.17 (0.25)	1.15 (0.26)
Psychosis^c^	1.31 (0.36)	1.22 (0.32)	1.21 (0.28)	1.17 (0.27)
Somatization	1.35 (0.33)	1.28 (0.29)	1.26 (0.27)	1.26 (0.29)
Environmental load^a^				
Zero	645 (21.35)	286 (23.29)	303 (11.89)	204 (9.23)
One	1711 (56.64)	694 (56.51)	1045 (41.01)	773 (34.98)
Two	556 (18.40)	210 (17.10)	980 (38.46)	958 (43.35)
Three	109 (3.61)	38 (3.09)	220 (8.63)	275 (12.44)

*The sample at T1 only included the younger members of the cohort; assessments at T0, T2 and T3 were based on the full sample. ^a^n (%); ^b^Self-report Symptom Checklist-90-R (SCL-90-R) symptom dimensions scores; ^c^paranoid ideation and psychoticism dimensions were combined to create a single “psychosis” dimension

**Table 2:** Characteristics of the population at different time points (NEMESIS-1)

	T0n (*n* = 7076)	T1n (*n* = 5618)	T2n (*n* = 4848)
	n (%)	n (%)	n (%)
Sex (male)	3299 (46.63)	2611 (46.48)	2255 (46.51)
Age^a^	41.16 (12.19)	42.09 (11.94)	44.19 (11.90)
Diagnostic categories^b^			
Anxiety disorders^c^	1423 (20.11)	430 (7.65)	332 (6.85)
Dysthymia	498 (7.04)	110 (1.96)	71 (1.46)
Major depression	1164 (16.45)	314 (5.59)	306 (6.31)
Manic episode	293 (4.14)	84 (1.50)	48 (0.99)
Psychosis^d^	295 (4.17)	72 (1.28)	45 (0.93)
Environmental load			
Zero	3171 (44.82)	2623 (46.69)	2285 (47.13)
One	2640 (37.31)	2173 (38.68)	1886 (38.90)
Two	961 (13.58)	670 (11.93)	553 (11.41)
Three or more	303 (4.28)	152 (2.71)	124 (2.56)

^a^Mean (standard deviation); ^b^binary lifetime diagnostic constructs according to the CIDI version 1.1 structured interview; ^c^includes panic disorder, agoraphobia, simple phobia, social phobia, generalized anxiety disorder, and obsessive–compulsive disorder; ^d^binary measure of any clinically relevant psychotic symptom

### Symptom connectivity in the EDSP

**Fig. 1:**
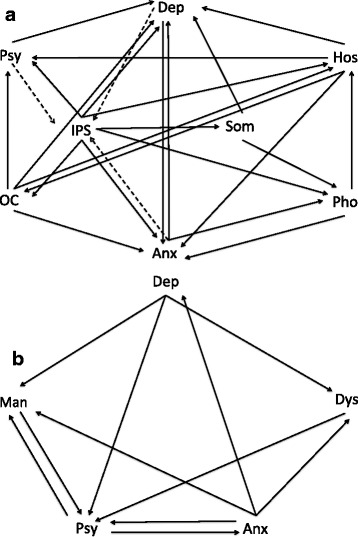
The impact of environmental exposure on connectivity across the symptom network. Fig. 1 (a) represents the EDSP sample. Fig. 1 (b) represents the NEMESIS-I sample. Arrows point out the direction of the association (from independent variable to dependent variable). Solid line indicates an increased association between symptom dimensions as a function of the degree of the environmental exposure. Dashed line indicates a reduced association between symptom dimensions as a function of the degree of the environmental exposure. Anx, Anxiety; Dep, Depression; Dys, Dysthmia; Hos, Hostility; IPS, Interpersonal Sensitivity; Man, Mania; OC, Obsessive-Compulsive; Pho, Phobic anxiety; Psy, Psychosis; Som, Somatization

All symptom dimensions were strongly connected to each other, and several significant interactions between symptom dimensions and environmental exposure score were found (Fig. 1a). With the exception of interpersonal sensitivity, for which reduced associations were apparent in the context of greater environmental exposure, connectivity between symptom dimensions increased with greater level of exposure to environmental risk factors (Table 3).

**Table 3:** The association between symptom dimensions as a function of exposure to environmental risk factors in the EDSP sample

Dependent	Independent	B (dimension)	B (environment)	B (interaction)
Depression	Anxiety	0.79^***^	NS	0.03^**^
	Psychosis	0.94^***^	NS	0.03^**^
	OCD	0.7^***^	−0.05^***^	0.05^***^
	Somatization	0.62^***^	NS	0.03^*^
	Phobia	0.93^***^	NS	NS
	Hostility	0.55^***^	NS	0.03^***^
	Sensitivity	0.63^***^	−0.05^***^	0.05^***^
Psychosis	Anxiety	0.62^***^	NS	NS
	Depression	0.58^***^	NS	NS
	OCD	0.52^***^	NS	0.01^*^
	Somatization	0.47^***^	NS	NS
	Phobia	0.71^***^	NS	NS
	Hostility	0.43^***^	−0.02^*^	0.02^**^
	Sensitivity	0.52^***^	−0.03^***^	0.03^***^
Anxiety	Depression	0.51^***^	−0.04^***^	0.02^**^
	Psychosis	0.67^***^	−0.03^**^	0.02^*^
	OCD	0.49^***^	−0.05^***^	0.03^***^
	Somatization	0.59^***^	NS	NS
	Phobia	0.73^***^	−0.06^***^	0.04^***^
	Hostility	0.39^***^	−0.04^***^	0.03^***^
	Sensitivity	0.41^***^	−0.04^***^	0.03^***^
OCD	Anxiety	0.78^***^	NS	NS
	Psychosis	0.88^***^	NS	NS
	Depression	0.73^***^	NS	NS
	Somatization	0.57^***^	NS	NS
	Phobia	0.94^***^	NS	NS
	Hostility	0.49^***^	NS	0.02^*^
	Sensitivity	0.54^***^	−0.04^***^	0.04^***^
Somatization	Anxiety	0.51^***^	NS	NS
	Psychosis	0.44^***^	NS	NS
	OCD	0.32^***^	−0.04^**^	0.03^**^
	Depression	0.35^***^	NS	NS
	Phobia	0.47^***^	NS	NS
	Hostility	0.28^***^	NS	NS
	Sensitivity	0.26^***^	NS	0.02^*^
Phobia	Anxiety	0.48^***^	−0.04^***^	0.04^**^
	Psychosis	0.51^***^	NS	0.02^*^
	OCD	0.40^***^	−0.05^***^	0.04^***^
	Somatization	0.35^***^	−0.04^**^	0.03^***^
	Depression	0.41^***^	−0.02^**^	0.02^**^
	Hostility	0.29^***^	NS	0.02^**^
	Sensitivity	0.32^***^	−0.04^***^	0.04^***^
Hostility	Anxiety	0.74^***^	NS	NS
	Psychosis	0.89^***^	NS	NS
	OCD	0.59^***^	−0.04^*^	0.02^*^
	Somatization	0.61^***^	NS	NS
	Phobia	0.81^***^	NS	NS
	Depression	0.69^***^	NS	NS
	Sensitivity	0.58^***^	−0.03^*^	0.02^*^
Sensitivity	Anxiety	0.86^***^	0.05^**^	−0.04^***^
	Psychosis	1.19^***^	0.05^***^	−0.05^***^
	OCD	0.73^***^	NS	NS
	Somatization	0.62^***^	NS	NS
	Phobia	0.99^***^	NS	NS
	Hostility	0.64^***^	NS	NS
	Depression	0.88^***^	0.04^***^	−0.05^***^

Dependent variables for each analysis were reported on the far left column, and next to those were independent variables. For each analysis examining connectivity between symptom dimensions as a function of exposure to environmental risk factors, unstandardized regression coefficients (B) for symptom dimension, environmental exposure, and interaction between environmental exposure and symptom dimension were provided, respectively. For example, the first row shows coefficients from the analysis investigating to what degree the exposure to environment moderated the impact of anxiety predicting depression

NS : non-significant, ^***^ *p* < 0.001, ^**^ *p* < 0.01, ^*^ *p* < 0.05

Environmental exposure increased associations of depression with other symptom dimensions except phobia. Similarly, significant interactions were found between the environmental exposure score and (i) interpersonal sensitivity, (ii) OC, and (iii) hostility in models predicting psychosis; and (i) hostility, and (ii) interpersonal sensitivity in models predicting OC. Regression analyses predicting anxiety revealed that associations with other symptom dimensions with the exception of somatization, increased as a function of the environmental exposure score.

Significant interactions were found between the environmental exposure score and (i) OC, and (ii) interpersonal sensitivity in models of somatization; and (i) OC, and (ii) interpersonal sensitivity in models of hostility. Regression analyses predicting phobia showed significant interactions between the environmental exposure score and each of the symptom dimensions. Regression analyses predicting sensitivity showed that associations between sensitivity and (i) anxiety, (ii) psychosis, and (iii) depression decreased as a function of the environmental exposure score.

### Symptom connectivity in the NEMESIS-1

All diagnostic domains were strongly connected with each other, and significant interactions between domains and the environmental exposure score were found (Fig. 1b). Regression analyses predicting major depression showed that only the strength of the association between major depression and anxiety disorders increased as a function of environmental exposure, whereas environmental exposure increased the strength of associations between psychotic symptoms and each of the diagnostic domains (Table 4). Regression analyses predicting anxiety disorders showed only the association between anxiety disorders and psychotic symptoms increased as a function of environmental exposure. Significant interactions were found between the environmental exposure score and (i) major depression, (ii) psychotic symptoms, and (iii) anxiety disorders in models predicting mania; as well as (i) major depression and (ii) anxiety disorders in models of dysthymia (Table 3).

**Table 4:** The association between symptom dimensions as a function of exposure to environmental risk factors in the NEMESIS-1 sample

Dependent	Independent	RD (dimension)	RD (environment)	RD (interaction)
Psychosis	Depression	0.04^***^	0.01^***^	0.04^***^
	Anxiety	0.03^***^	0.01^***^	0.05^***^
	Mania	0.12^***^	0.01^***^	0.07^**^
	Dysthymia	0.05^**^	0.02^***^	0.04^**^
Depression	Psychosis	0.23^***^	0.05^***^	NS
	Anxiety	0.21^***^	0.04^***^	0.05^***^
	Mania	0.35^***^	0.05^***^	NS
	Dysthymia	0.55^***^	0.05^***^	NS
Anxiety	Psychosis	0.29^***^	0.05^***^	0.05^*^
	Depression	0.29^***^	0.04^***^	NS
	Mania	0.41^***^	0.05^***^	NS
	Dysthymia	0.37^***^	0.05^***^	NS
Mania	Psychosis	0.12^***^	0.02^***^	0.08^***^
	Anxiety	0.04^***^	0.01^***^	0.06^***^
	Depression	0.05^***^	0.01^***^	0.03^***^
	Dysthymia	0.06^***^	0.02^***^	NS
Dysthymia	Psychosis	0.10^***^	0.02^***^	NS
	Anxiety	0.10^***^	0.01^***^	0.03^***^
	Mania	0.15^***^	0.02^***^	NS
	Depression	0.20^***^	0.01^***^	0.02^*^

Dependent variables for each analysis were reported on the far left column, and next to those were independent variables. For each analysis examining connectivity between symptom dimensions as a function of exposure to environmental risk factors, risk differences (RD) for symptom dimension, environmental exposure, and interaction between environmental exposure and symptom dimension were provided, respectively. For example, the first row shows risk differences from the analysis investigating to what degree the exposure to environment moderated the impact of depression predicting psychosis

NS: non-significant, ^***^ *p* < 0.001, ^**^ *p* < 0.01, ^*^ *p* < 0.05

## Discussion

This study investigated to what degree connectivity between symptom dimensions and diagnostic categories is conditional on environmental exposures known to be associated with mental ill health, particularly psychosis. The principal findings were: (i) symptom dimensions and related diagnostic categories were strongly associated with each other; (ii) environmental exposures increased the level of connectivity.

### Psychopathology as a network of symptoms

Our findings showing strong connectivity across symptom dimensions and related binary diagnostic constructs in two independent population cohorts confirm previous results [9], and provide additional evidence for the conceptualization of psychopathology as a network of mutually interacting symptoms [3–6]. Several studies in general population cohorts have shown that symptom domains do not vary in isolation but are interconnected, even before the emergence of a distinct clinical state, and that interactions between symptoms, including transdiagnostic ‘contiguous symptoms’ (e.g. hallucinations x delusions) and ‘disjointed symptoms’ (e.g. affective dysregulation x paranoia) predict transition to a more severe mental state [39–41]. Micro-level research of psychopathology at the level of momentary experiences in the flow of daily life using the Experience Sampling Method (ESM) has demonstrated that there is a continuous dynamic interplay between momentary mental states, with increasing connectivity between momentary states (paranoia, positive, and negative affect) predicting increased severity [42] and onset of need for care [43]. Similarly, an investigation of the underlying dynamic network structure of psychopathology revealed that mental states of individuals with psychosis or depression were more connected than those of healthy controls, with more feedback loops across negative momentary mental states (e.g. “insecure”, “suspicious”) [44]. There is some evidence that an alteration in the pattern of mood dynamics, marked by increased connectivity across the network, may give rise to an unstable condition, or tipping point, where even a subthreshold stimulus may provoke an abrupt transition to clinical depression [45].

### The impact of environmental exposures on the psychopathology network

The findings indicating increased symptom connectivity as a function of the level of environmental exposure score lend further support to previous findings showing that exposure to environmental risk factors (trauma, urbanicity, cannabis) additively increase psychosis expression in affective spectrum disorder in a dose–response fashion [9]. Smeets and colleagues demonstrated, both in general population samples [46] and in those at high genetic risk (siblings and parents of patients) [8] that rates of co-occurrence of hallucinations and delusions are moderated by environmental exposure (cannabis use and childhood trauma). Another recent study in the general population showed that when symptoms were grouped together to form a connected symptom domain, association with childhood trauma became significantly stronger than when symptoms were analysed in isolation [7]. Similarly, an ESM study showed that childhood adversity moderated the impact of negative affect on paranoia levels [47]. Furthermore, meta-analytic work has shown that environmental exposures increase the way psychotic symptoms impact on to themselves over time – i.e. the rate of persistence or the rate of transfer over time. Thus‚ greater level of exposure to childhood trauma predicts increased persistence of psychotic experiences [48].

### Strengths and limitations

The major strength of this study is the analysis of hypothesized psychopathology networks at levels of both continuous symptom dimensions and related binary diagnostic constructs in two independent large, representative population cohorts assessed with standardized clinical instruments administered by trained interviewers, thus increasing the generalizability of our findings. However, there are several limitations that should be taken into consideration. First, observations from all time points were combined in the ‘long format’–cross-sectional in nature. This is a practical solution at the current stage, given the lack of a sufficiently large dataset with intensive repeated assessments over an extended time frame, allowing for sequential analysis of the association between environmental exposures and symptom connections. While cross-sectional analysis increases reliability, it may not be the optimal approach to elucidate causal associations between environmental exposures and symptom connectivity over time. Future studies should therefore aim to investigate large cohorts with more frequent and fine-grained follow-ups in order to gain more insight into the dynamic formation of psychopathology networks under the influence of environmental exposures. Second, based on previous evidence indicating that environmental factors additively reinforce each other, possibly impacting on the same underlying mechanism, environmental risk factors were combined to construct a cumulative environmental risk load. The use of a score reflecting combined environmental impact increased statistical power and reduced type I error. On the other hand, while environmental factors may not all contribute equally to the cumulative environmental impact, similar weights were assigned to each environmental factor, which may have produced a degree of imprecision to the results.

## Conclusions

Evidence indicates that environmental risk factors impact on connections between symptoms in a causal network, increasing connectivity, thus adding to tipping the balance towards transition from the initial set of subthreshold symptoms to a more severe mental state and a more distinct clinical syndrome requiring treatment.

Under the framework of connected symptoms in a network, future research may focus on the divergent and interactive impacts of vulnerability factors (environmental and genetic) on pattern formation across symptoms and mental states, and investigate whether there are specific patterns in the network that may be useful to differentiate and predict psychopathology at disorder and symptom levels. Despite high cost, the optimal research design would require a large enough cohort with a longitudinal design to assess psychopathology (both at micro and macro levels) and environmental exposures over a longer period of time, spanning the period of transition from a normal to a clinical state e.g. in late adolescence and early adulthood. A more practical approach to understanding changes of network patterns leading to progression to a clinical syndrome may be to use a sibling-case–control design providing the opportunity to investigate the symptom network at different levels of genetic and non-genetic susceptibility [49].

## Abbreviations

EDSP study, The Early Developmental Stages of the Psychopathology; ESM, the Experience Sampling Method; NEMESIS-1, The Netherlands Mental Health Survey and Incidence Study; OC, obsessive-compulsive; SCID-I, the Structured Clinical Interview for Diagnostic and Statistical Manual of Mental Disorders, Fourth Edition; SCL-90-R, the Self-report Symptom Checklist-90-R
